# Multicentric reticulohistiocytosis after severe COVID-19 infection

**DOI:** 10.1016/j.jdcr.2023.01.014

**Published:** 2023-01-28

**Authors:** Colin M. Kincaid, Ajay N. Sharma, Justin D. Arnold, Luke Horton, Bonnie A. Lee, Natasha A. Mesinkovska

**Affiliations:** Department of Dermatology, University of California, Irvine, Irvine, California

**Keywords:** COVID-19, histiocytosis, multicentric reticulohistiocytosis, IL, interleukin, MRH, multicentric reticulohistiocytosis, TNF, tumor necrosis factor

## Introduction

Multicentric reticulohistiocytosis (MRH) is a rare condition characterized by a papulonodular cutaneous eruption and an erosive polyarthritis.^1^ While the pathophysiology of MRH is poorly understood, it is considered a systemic inflammatory response to autoimmune conditions, malignancy, or rarely, infections.^2^ Many inflammatory skin conditions have previously been reported as sequalae of COVID-19.^3^ Herein, we report our experience treating a patient who developed MRH after a severe COVID-19 infection.

## Case report

A 56-year-old Caucasian female with history of seronegative hypothyroidism presented to the dermatology clinic with a 3-month history of progressive erythematous patches on her face and chest, a papular eruption on her dorsal hands and fingers, and severe weakness and polyarthralgia of her hands and knees that limited her mobility and activities of daily living. The symptom onset was approximately 1 month after a COVID-19 infection requiring admission to the intensive care unit. During her hospitalization, she experienced acute hypoxemic respiratory failure, bilateral deep vein thromboses, and ischemic acute tubular necrosis requiring hemodialysis, atrial fibrillation, and polyneuropathy with bilateral foot drop which left her with a significant gait impairment. The patient was treated with remdesivir and dexamethasone for her COVID-19 infection. Additional review of her medical history revealed stable micro-prolactinoma diagnosed 7 years prior, exercise induced asthma, and anxiety, but no prior history of joint pain. Her medications prior to hospitalization included cabergoline, levothyroxine, albuterol inhaler, and alprazolam. Family history was significant for psoriasis vulgaris, celiac disease, and triple negative breast cancer in her daughter.

**Fig 1:**
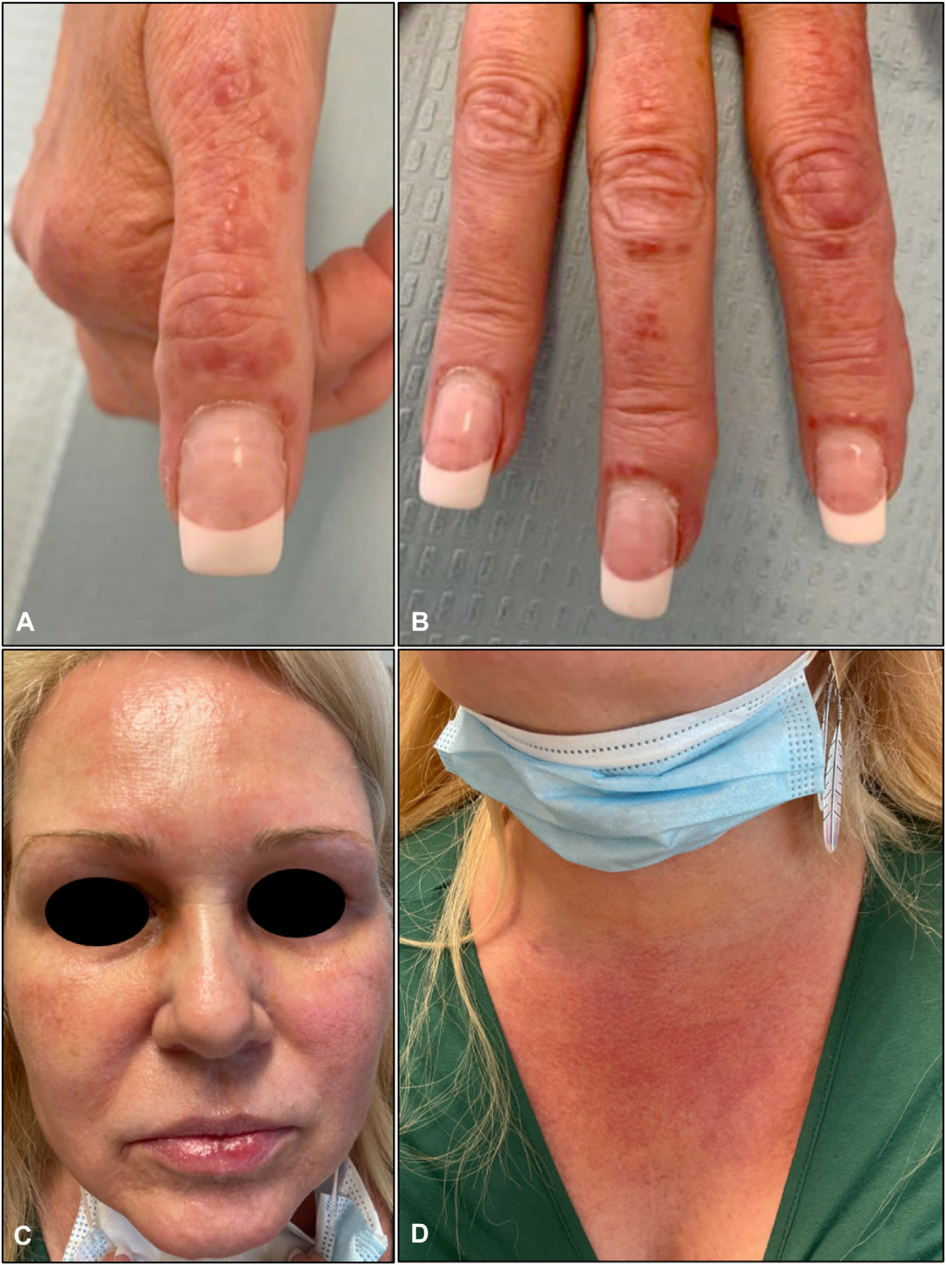
**A,** Erythematous papules on the dorsal hand. **B,** Characteristic periungual distribution of papules with “coral bead” appearance. **C,** Erythematous patches on the bilateral cheeks, nasal sidewall, and forehead. **D,** Erythematous patch on the chest.

Clinical examination revealed numerous 2 to 3 mm tender, skin-colored to pink papules on her bilateral dorsal hands and feet with a ring-like arrangement perinugually on her digits (Fig 1, *A* and *B*). Ill-defined faint pink patches were noted on her cheeks, nasal sidewall, forehead, and chest (Fig 1, *C* and *D*). Her exam was also significant for diffuse hair thinning of the scalp, and severe symmetric range of motion impairment in the knees and fingers with inability to form a fist. No abnormalities were appreciated on her tongue or oral mucosa.

**Fig 2:**
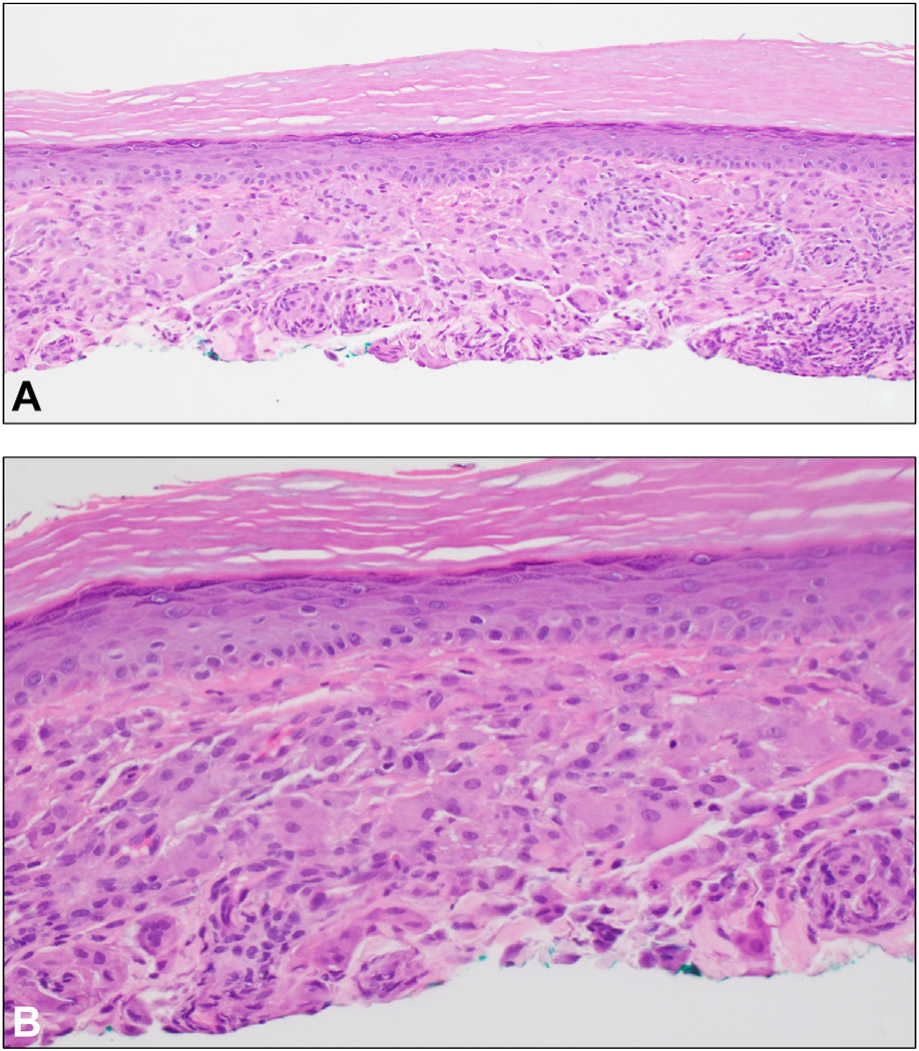
Histopathologic images of a biopsy specimen from papule on the finger (hematoxylin-eosin) (**A**) dermal collection of mono- and multinucleated histiocytes. **B,** Multinucleated histiocytes with abundant “ground glass” cytoplasm. (**A** and **B,** Hematoxylin-eosin stain; original magnifications: **A,** ×100; **B,** ×200.)

On histology, biopsy of a papule on the right dorsal third finger revealed dermal collections of mono- and multinucleated histiocytes with very finely granular, almost homogenized two-toned cytoplasm (often referred to as “ground glass”), consistent with MRH (Fig 2).

Following diagnosis, the patient underwent extensive rheumatologic workup (markers associated with connective tissue diseases, rheumatoid arthritis, and celiac disease) as well as tuberculosis screening which were unremarkable. Cancer screening including computed tomography (chest, abdomen, and pelvis), colonoscopy, endoscopy, mammogram, breast ultrasound, gynecologic exam, protein electrophoresis, flow cytometry, kappa lambda free light chain, carcinoembryonic antigen, cancer antigen 19-9, and cancer antigen 27-29 was unremarkable.

The treatment of MRH with the goal of skin and joint symptom improvement proved challenging. Initially, the patient mildly responded to prednisone 20-60 mg and hydroxychloroquine 200 mg daily, but symptoms returned upon steroid tapering. After 2 months, methotrexate 25 mg per os weekly was added with minimal improvement in her arthralgias and the cutaneous eruption. Given several reports of successful treatment with tumor necrosis factor (TNF)-alpha inhibitors,^4^ the patient was transitioned to adalimumab 40 mg subcutaneous every other week with methotrexate 25 mg per os weekly, which has resulted in significant improvement in the erythema and induration of skin papules after 3 months of dual therapy. Additionally, she has begun to regain mobility of her fingers of hands with residual arthralgias and weakness.

## Discussion

MRH remains a poorly understood multisystem disease, mostly due to its rarity as only approximately 200 cases have been reported in the literature since 1937 when the condition was first described.^1^^,^^4^ Cutaneous lesions may occur virtually anywhere on the body, with pathognomonic signs including periungual distribution of papules (“coral beads”) or vermicular lesions surrounding the nostrils.^5^ Additional features such as periungual telangiectasias, pruritus, xanthelasma, oral mucosal nodules, and dermatomyositis-like photodistributed erythema may be present.^4^ The erosive polyarthritis associated with MRH is symmetrical and may progress to severe joint destruction leading to significant functional disability.^1^^,^^4^

The pathophysiology of this condition is puzzling and based primarily on its comorbid associations with underlying malignancy and autoimmune diseases. Malignancy has been reported in 15% to 31% of MRH cases, with no clear predilection for organ system involved.^2^ Various malignancies including breast, stomach, ovary, penis, cervix, endometrium, pancreas, lung, skin, and hematologic have all been reported in association with MRH.^4^, ^5^, ^6^ Thus, a broad malignancy screening beyond what would be considered age-appropriate is often performed in patients diagnosed with MRH. A wide variety of autoimmune diseases, including thyroid disorders, have also been reported in up to 15% cases of MRH,^2^ suggesting that the inflammatory milieu triggers the MRH disease process driven by proliferation of histiocytes.^7^ The prominent staining of TNF, interleukin (IL)-12, and IL-1b on synovial biopsies from affected joints in MRH provides insight into cytokines that play a role in the pathogenesis of MRH.^8^

The patient in our case underwent extensive malignancy screening and autoimmune serology workup, all of which were unremarkable, with exception of stable micro-prolactinoma and hypothyroidism. The temporal relationship between MRH symptoms and the severe COVID-19 infection provides a speculative clue into the etiology. In other cases of new-onset autoimmune conditions following COVID-19, symptoms have been reported to onset approximately 1 month post-infection as was observed in this case.^9^ In patients requiring intensive care unit admission for severe COVID-19 infection, highly elevated levels of circulating proinflammatory cytokines including IL-1a, IL1-b, IL-6, IL-18 and TNF have been observed.^10^ It is plausible that the COVID-19 inflammatory response can in turn induce histiocyte proliferation and aberrantly lead to development of MRH. Additional studies to assess the inflammatory milieu which may both induce and sustain MRH are needed to better understand the etiology of this debilitating condition and to guide therapeutics.

## Conflicts of interest

None disclosed.
